# Connexin 26 (*GJB2*) mutation in an Argentinean patient with keratitis-ichthyosis-deafness (KID) syndrome: a case report

**DOI:** 10.1186/s12881-016-0298-y

**Published:** 2016-05-04

**Authors:** Viviana Karina Dalamón, Paula Buonfiglio, Margarita Larralde, Patricio Craig, Vanesa Lotersztein, Keith Choate, Norma Pallares, Vicente Diamante, Ana Belén Elgoyhen

**Affiliations:** Instituto de Investigaciones en Ingeniería Genética y Biología Molecular “Dr. Héctor Torres”-(INGEBI), Consejo Nacional de Investigaciones Científicas y Técnicas (CONICET), Vuelta de Obligado 2490. (1428) Ciudad Autónoma de Buenos Aires, Buenos Aires, Argentina; Servicio de Dermatología Pediátrica, Hospital Ramos Mejía, Ciudad Autonoma de Buenos Aires, Argentina; Departamento de Dermatología, Hospital Alemán, Ciudad Autonoma de Buenos Aires, Argentina; Departamento de Química Biológica e Instituto de Química y Fisicoquímica Biológicas, Universidad de Buenos Aires, Consejo Nacional de Investigaciones Científicas y Técnicas (CONICET), Ciudad Autonoma de Buenos Aires, Argentina; Servicio de Genética, Hospital de Clínicas “José de San Martín”, Ciudad Autonoma de Buenos Aires, Argentina; Dermatology Yale University School of Medicine, New Haven, USA; Instituto Superior de Implantes Cocleares “Dr. Vicente Diamante”, Ciudad Autonoma de Buenos Aires, Argentina; Departamento de Farmacología, Facultad de Medicina, Universidad de Buenos Aires, Ciudad Autonoma de Buenos Aires, Argentina

**Keywords:** *GJB2*, Mutations, KID syndrome, Connexin, Deafness, p.Asp50Asn

## Abstract

**Background:**

Keratitis-Ichthyosis-Deafness (KID) syndrome is a rare condition characterized by pre-lingual sensorineural deafness with skin hyperkeratinization. The primary cause of the disease is a loss-of-function mutation in the *GJB2* gene. Mutations in Argentinean patients have not been described.

**Case presentation:**

We studied a 2 year-old boy with bilateral congenital sensorineural deafness with dry skin over the entire body, hypotrichosis of the scalp, thin and light-blond hair. Analysis of the *GJB2* gene nucleotide sequence revealed the substitution of guanine-148 by adenine predicted to result in an Asp50Asn amino acid substitution.

**Conclusion:**

This is the first KID report in a patient from Argentina. This *de novo* mutation proved to be the cause of keratitis-ichthyosis-deafness syndrome (KID-syndrome) in the patient, and has implications in medical genetic practice.

## Background

KID syndrome (keratitis–ichthyosis–deafness) is an ectodermal disorder characterized by the association of keratitis and hearing loss (OMIM 148210). It belongs to the clinically and genetically heterogeneous group of erythrokeratodermias. It is usually characterized by sensorineural hearing loss accompanied with erythrokeratoderma, hyperkeratosis of the palms and soles and recurrent bacterial or fungal infections, and could be associated with photophobia and corneal vascularization [1–3]. The skin lesions, described as erythrokeratoderma occurs occasionally with scarring alopecia and are predominantly on the face, palms and soles, with a typical reticulated pattern that is often defined as leather-like. Additional features are corneal epithelial defects, including scarring and neo-vascularization that can result in progressive decline of visual acuity and may eventually lead to blindness. It has been described that KID syndrome is associated with an increased susceptibility for squamous cell carcinomas of tongue and skin in at least 10 % of patients, apparently due to p53 loss in the lesions [4–7].

To date, less than 100 cases of KID syndrome, the majority of which are sporadic and of autosomic dominant inheritance, have been described in the world literature. Some familial forms have been reported [4, 8–10]. KID syndrome is caused by heterozygous missense mutations in *GJB2*, the gene encoding connexin 26 (Cx26). Cx26 is a protein of 26-kDa that forms the intercellular channels of gap junctions and plays part in a variety of biological functions such as cell growth, tissue homeostasis and development. In the epidermis, gap junctions appear to play a role in the coordination of keratinocyte growth and differentiation, whereas in the sensory epithelia of the inner ear they mainly regulate the potassium recycling during auditory transduction [11, 12]. Several observations suggest that changes in the proliferation and differentiation pathway of keratinocytes correspond with a switch of the pattern of Cx expression in skin. Dominant mutations in Cx26 have been described in several syndromic deafness associated with skin diseases with varying phenotypes, including KID, palmoplantar keratoderma with deafness, Bart-Pumphrey syndrome, Vohwinkel syndrome, and Hystrix-like ichtyosis-deafness syndrome [13]. Genetically, all of these disorders result from mutations in *GJB2*, but the nature of each disease depends on the particular mutation detected. Autosomal recessive hearing loss without skin involvement (called non syndromic) is mostly due to the loss of channel function that produce the alteration of cochlear intercellular communication [14–16]. In syndromic hearing loss instead, mutant connexins are supposed to acquire novel properties that alter epidermal differentiation [17]. Several functional analyses of specific Cx26 mutations have been carried out to decipher which are the molecular mechanisms underlying the different phenotypes observed in these different disorders. Among them, Vohwinkel syndrome was the first of the known skin diseases to be attributed to a mutation in *GJB2* (c.G196C leading to p.Asp66His) [18]. Most cases of KID syndrome arise from a recurrent missense mutation, c.148G > A (p.Asp50Asn), and it has been reported in patients worldwide from different ethnic groups [19–23]. In addition to this recurrent mutation, a few others have been now described in KID patients, such as p.Gly11Arg, p.Gly12Arg, p.Asn14Tyr, p.Ser17Phe, p.Ala40Val, p.Gly45Glu, p.Asp50Tyr and p.Gly54Glu [20, 24–27]. Most of the patients are sporadic cases, but there have been reports of families where the illness displays a dominant transmission pattern [20, 28, 29].

Cxs share a common pattern of structural motifs, which includes four transmembrane domains (TM1 to M4), two extracellular (E1, E2) and three cytoplasmic domains: the amino-terminus, a cytoplasmic loop and the carboxy terminus domain (NT, CL and CT respectively). The membrane spanning and the extracellular domains are highly conserved and the main differences between Cxs are found in their C-terminal tails [24]. The interaction of six connexins leads to the formation of an hemichannel called connexon, a functional unit across the plasma membrane. Connexons of two opposing cells interact with each other through their extracellular portions (E1 and E2), forming a channel that is the basic unit of the functional gap junction. This type of connection allows a rapid exchange of different molecules between two connected cells: small ions, secondary messengers and metabolites [24–26, 28]. In the case of KID syndrome, all pathogenic mutations were described clustering in regions coding for the first extracellular domain and the NH2-terminal of Cx26, implying common functional defects.

In this work, we identified a clinical case of KID syndrome with mutation p.Asp50Asn in *GJB2*, associated to sensorineural hearing loss and several skin alterations. Even though this mutation has already been described in other populations, this is the first report in Argentina and it contributes to the general knowledge of the pathology.

## Case Presentation

We studied a male patient with KID syndrome with unrelated healthy parents and a normal 46XY karyotype. The diagnosis was based on dermatological and hearing evaluations. At birth he presented skin alterations and hearing impairment. Diagnosis was confirmed by molecular analysis of the *GJB2* gene. Written consent was obtained from his parents.

### Clinical data

The patient was born at term, after an uneventful pregnancy and normal delivery. The parents were non-consanguineous and there was no family history of a similar condition.

**Fig. 1:**
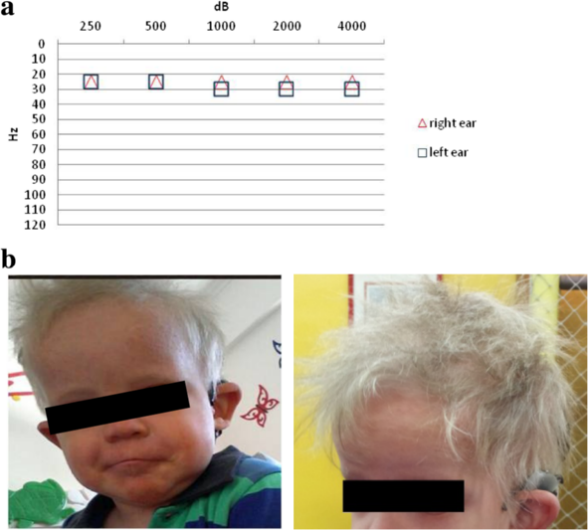
Illustration and audiometry of KID Argentinean case. **a** Audioprofile of the patient after cochlear implantation showing good outcome. **b** The scalp hair was thin, sparse and light-blond. He had aged facial appearance and hypotrichosis

Auditory features: the child had bilateral, profound, prelingual hearing loss. It was detected by Transient Evoked Otoacoustic Emissions (TEOAEs) at birth and diagnosed by subjective and objective tests. He was implanted bilaterally at 18 months with a Nucleus 24 Cochlear implant system. He had normal cochleae, with full insertion of the electrode array and no surgical complications. The speech processor map was obtained via behavioral observation and play conditioned responses. Initial tune-up and control was done during the followed days. Auditory sensations were produced in the child with the activation of all electrodes of the array. Then, re-mapping was done each month during the first 3 months, every 3 months during the first year and every 4 months during the second year, and resulted in a very good outcome (Fig. 1a). The child achieved speech detection and environmental sound awareness, showing auditory improvement in speech perception through specific tests (Latin American Protocol) and in it-MAIS and MAIS Scales according to the parents’ opinion. The evolution of pre and post cochlear implantation was evaluated using the Latin America Protocol for CI (Cochlear Corp) using Free Field audiometry with warble tones and speech perception tests.

At the time of writing this report the patient was 3 years old and has a history of chronic external otitis, and had two episodes of conjunctivitis.

Dermatological features: At birth he presented generalized thickening, dryness and scaling of the skin and alopecia. Keratitis was noticed at 6 months, compatible with cutis laxa. Physical examination revealed a generalized thickened skin and xeroderma, palmoplantar keratoderma and rippled hyperkeratotic plaques on the knees and elbows, as well as in the free edge of eyelids, nostrils and in the external auditory canal. -yellowish hyperqueratotic. The palms and soles showed hyperlinearity and mild hyperkeratosis. The scalp hair was thin, sparse and light-blond (Fig. 1b). He also had hypohidrosis. No alteration on mucosa, teeth or nails were found. He had aged facial appearance and hypotrichosis (sparse of eye lashes and eyebrows). His psychomotor development was normal. Compared to other described KID patients his phenotype was mild and his evolution over time was extremely favorable.

### Mutation analysis of *GJB2*

Genomic DNA was obtained from the patient’s and his relatives’ peripheral blood samples by standard techniques. All polymerase chain reactions were performed using the same protocol.

The PCR reaction mix contained: 200 μM dNTPs, 1.5 mM MgCl_2_, 20 mM Tris-ClH (pH8), 50 mM KCl and 1U Taq polymerase (Invitrogen, Life Technologies, Sao Paulo, Brazil) in a final volume of 25 μl. The reaction was carried out for 30 cycles with annealing at 60° for 30 s, extension at 72° for 30 s, denaturation at 94° for 40 s, with an initial denaturing step at 95° for 5 min and a final extension step of 72° for 5 min using a Bio-Rad PTC200 thermal cycler (Hercules, California, USA). PCR products were run on a 2 % agarose gel and stained with SyBr Safe to confirm fragment size (Invitrogen, Life Technologies, Eugene, Oregon, USA). PCR products were then purified from the remaining nucleotides and primers using QIAquick PCR purification Kit (Qiagen, GmbH, Hilden, Germany) according to the manufacturer’s protocol. Bi-directional DNA sequencing was performed on an automatic sequencer (3730xl DNA Analyzer, Applied Biosystems, Foster City, CA, USA). The sequence trace was aligned to the wild type sequence of the *GJB2* gene (NCBI accession number NG_008358.1) using the NCBI interface (http://www.ncbi.nlm.nih.gov/Blast.cgi).

Primers used for amplification were: for non-coding exon 1 of GJB2 gene: (Cx26E1-F) 5′CAGTCTCCGAGGGAAGAGG and (Cx26E1-R) 5′AAGGACGTGTGTTGGTCCAG; for coding exon 2 of GJB2 (Cx26E2F) 5′GAAGTCTCCCTGTTCTGTCCT and (Cx26E2R) 5′TCTAACAACTGGGCAATGC; for mitochondrial gene MT-RNR1 (Mt12S-F) 5′GCAAACCCTGATGAAGGCTA and (Mt12S-R) 5′GCGCCAGGTTTCAATTTCTA; for detection of the two big deletions in gen GJB6 the protocol detailed in del Castillo, et al [25].

### Sequence alignment and molecular modeling

The evolutionary conservation of the Cx26 mutated residue was assessed by performing a multiple sequence alignment of different mammalian Cx26 sequences, which were retrieved from the Ensembl database (http://www.ensembl.org). The Mega5 version5 (http://www.megasoftware.net/) software was used for generating the sequence alignment. The pathological nature of the substitution was confirmed by in silico- analysis, using PolyPhen (http://genetics.bwh.harvard.edu/pph2/) and SIFT (http://sift.jcvi.org/) informatic tools. Structure/function analysis was performed by modeling the p. Asp50Asn mutant with programs Spdbviewer [26] (http://www.expasy.org/spdbv/), SCWRL [27, 28] (http://www1.jcsg.org/scripts/prod/scwrl/serve.cgi) and FOLDX [29] (http://foldx.crg.es/), using the pdb structure 2ZW3 as template. The figure of the structural model was made with the program VDM (http://www.ks.uiuc.edu/Research/vmd/) [30].

## Results

**Fig. 2:**
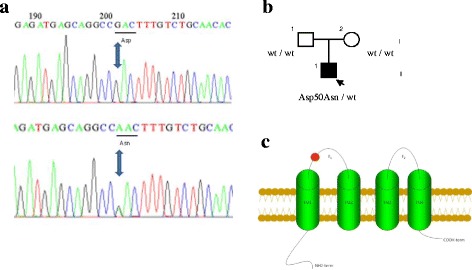
Genetic testing. **a** Chromatogram of the patient’s *GJB2* sequence shows an G → A transition (c.G148A), predicting a of Asn by Asp substitution in codon 50 (p.Asp50Asn). Upper diagram shows the wild type sequence and lower image the mutated version in the patient. **b** Patient’s family pedigree. **c** Schematic diagram of connexin 26 protein in the plasma membrane. *Red circle* marks the position of detected mutation which is localized in the E1 extracellular domain. IC: intracellular domain, TM: transmembrane domain, EC: extracellular domain

The patient harbored an adenine 148 for guanine transition (c.G148A), leading to an asparagine for aspartic acid substitution at position 50 of Cx26 (p.Asp50Asn) (Fig. 2a). Mutation p.Asp50Asn was not present in more than 800 patients with hearing defects but no skin disorder nor in 100 healthy controls of Argentinean origin.

DNA from the patient was also screened for mutations in the basal promoter, exon 1 and the donor splice site of the *GJB2* gene, as well as for the deletions del(GJB6-D13S1830) and del(GJB6-D13S1854) in the *GJB6* gene. As maternal inheritance could not be excluded, the presence of m.A1555G and m.C1494T mutations in the mtDNA was assessed. No further mutations were detected in this patient. The parents did not harbor the mutation, revealing a *de novo* mutation (Fig. 2b).

**Fig. 3:**
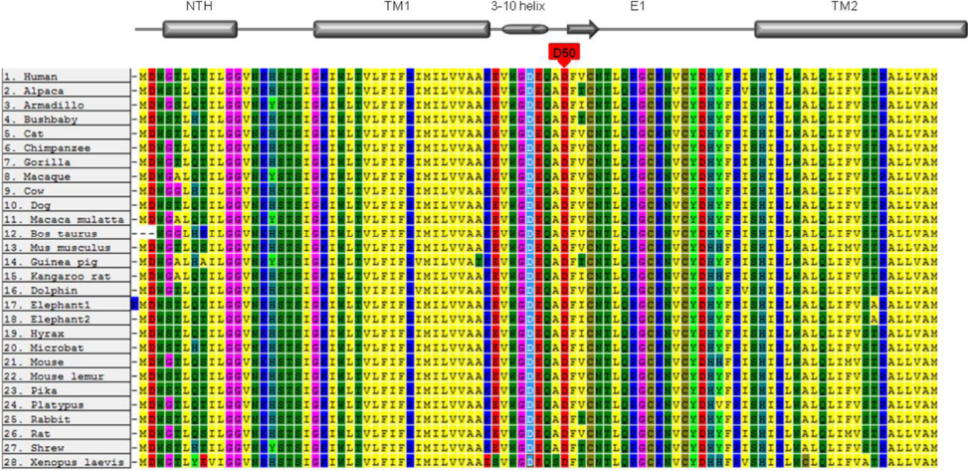
Connexin alignment. The Cx26 sequences from different species were aligned using the Mega5 program, in order to investigate the evolutionary conservation of the aspartic residue located in codon. The multiple alignments revealed a total conservation of this residue across all species, suggesting that this residue is crucial for the protein functionality

Functional analyses of the p.Asp50Asn mutation performed in the *Xenopus laevis* oocyte expression system and immunohistochemical analyses in skin biopsies have shown alterations in connexin 26 function. Residue Asp50 is predicted to be located at the highly conserved extracellular domain (E1) of Cx26 (Fig. 2c), a region suggested to play a crucial role in voltage gating and connexon-connexon interactions. The complete conservation of Asp 50 shown in the alignment of Cx26 proteins form 28 different species (Fig. 3), reinforces a key role of this residue in protein function [19, 23, 31]. *In silico* analysis using PolyPhen and SIFT informatics tools confirms that the mutation is either damaging or deleterious, respectively.

**Fig. 4:**
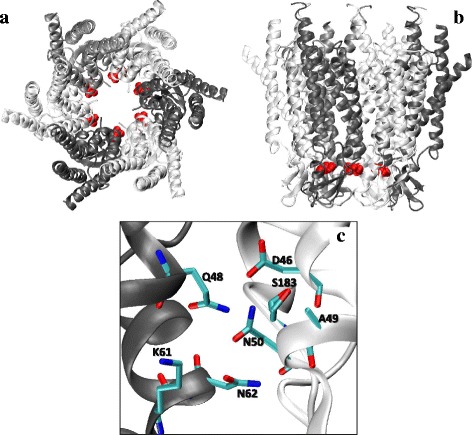
Modeling of the Asp50Asn mutant (D50N). In order simplify the figure the one letter nomenclature was used for amino acids. The figure was obtained using the software VMD. **a** and **b** show the location of D50 (*red*) in the structure of the channel. **c** shows the theoretical structural model of the D50N mutant. The direct contact interactions on a radial distribution of 5 Å for each mutant are shown in the corresponding panels. D50 makes contacts with D46, A49 and S183 of the same chain, and Q48, K61 and N62 of a contiguous chain. This residue is located in an internal region of the channel enriched in acidic residues (D46 and E47) balanced with basic residues (R184, K61, and K188). The mutation D50N decreases the negative charge of this environment as well as alters the core of the inter-protomer interactions. The theoretical model shows that the D50N mutation breaks a salt bridge that exists between D50 and K61 and produces a hydrogen bond between the side chain of N50 and D46

In order to further analyze the molecular perturbations introduced by the p.Asp50Asn mutation we modeled the wild type and mutant proteins. Asp 50 faces the interior of the pore and participates creating a long, negatively charged path, near the surface of the extracellular membrane. Panel a and b of Fig. 4 show the location of Asn50 in the structure of the channel (D50 in red). Panel c of Fig. 4 shows the theoretical structural model of the Asp50Asn mutant (in figure shown as D50N). The direct contact interactions on a radial distribution of 5 Å for each mutant are shown in the corresponding panels. Modeling predicts that Asp50 makes contacts with Asp46, Ala49, and Ser183 of the same chain, and Gln48, Lys61, and Asn62 of a contiguous chain (Fig. 4c). Asp50 is located in an internal region of the channel enriched in acidic residues (Asp46 and Glu47), balanced with basic residues (Arg184, Lys61, and Lys188). The mutation Asp50Asn decreases the negative charge of this milieu, breaks a salt bridge between Asp50 and Lys61 and produces a hydrogen bond between the side chain of Asn50 and Asp46. Collectively, bioinformatics analyses strongly confirm the pathogenic nature of the D50N mutation.

## Conclusions

This is the first report of an Argentinean patient with KID syndrome due to the p.Asp50Asn mutation in the *GJB2* gene. More than eight hundred patients with deafness have been studied in our laboratory in Argentina. Nevertheless, this is the first patient with a skin associated phenotype. We and others have reported 223 different mutations in *GJB2* and *GJB6* associated with hearing impairment but not with skin abnormalities in Argentina [32–35]. Deafness-associated *GJB2* mutations (coding Cx26) can be classified into two groups: those that produce non-syndromic deafness and mutations that produce hearing loss associated with other signs such as skin alterations, ocular, renal or thyroid disorders (syndromic deafness). Syndromic forms of hearing impairment associated with skin disorders are of autosomal dominant inheritance, and the skin affections range from mild to severe. In addition to KID, other syndromes include Bart–Pumphrey syndrome, Vohwinkel syndrome, palmoplantar keratoderma, and hystrix-like ichthyosis deafness syndrome [36]. It has been proposed that *GJB2* mutations causing autosomal recessive non-syndromic hearing loss seems to be ethnically specific, probably based on founder effects, contrary to *GJB2* mutations related to syndromic hearing loss that seem not to be population specific [19, 37].

KID patients with the p.Asp50Asn mutation generally present profound deafness with complete or incomplete alopecia, most of them have some visual affection, recurrent bacterial or fungal skin infections, scalp, foot and/or tongue carcinomas, and different degrees of inflammatory nodules, hyperkeratosis, erythrodermia and skin lesions [19, 38]. The patient presented in this work had the classic phenotypic triad of KID syndrome including erythrokeratoderma, keratitis, and profound bilateral sensorineural hearing loss. Nevertheless, when compared to other KID patients his phenotype was mild and his evolution over time was extremely favorable.

Different *GJB2* mutations result in different functional impairments at the cellular level (e.g., docking, trafficking, or gating defects), which could result in different clinical phenotypes. The amino acid substitution in the p.Asp50Asn, occurs in the highly conserved first extracellular loop of Cx26, which is crucial for voltage gating and connexon-connexon interactions [39]. It is difficult to explain the variety of clinical findings and the range of disease progression seen in KID patients bearing the same Asp50 mutation. This further supports the lack of a straightforward genotype–phenotype correlation in patients with connexin mutations, not only in KID syndrome but also in non-syndromic hearing deafness [33, 40, 41].

Our and other results thus indicate that the Cx26 mutations associated with hearing loss in syndromic pathologies might affect cell functionality by much more complex mechanisms than a specific mutation, which include genetic background of the patient, cell-dependent effects, or interactions with other mutated genes producing cell survival modifications.

Asp50 is a pore-lining residue and would stabilize the open state of Cx26 hemichannels by interacting with positively residues in the adjacent connexin subunit. The negative charge of the aspartic residue would influence open hemichannel properties [42]. Expression studies have demonstrated that mutation Asp50Asn alters gap junction channel function causing aberrant Cx26 hemichannel openings, possibly as a result of its dual role as a component of an intersubunit complex and a pore-lining residue in the extracellular region of the hemichannel [42, 43]. According to crystal structure, each helix in a protomer would contribute to an aromatic cluster in the groove between two adjacent protomers. Alignment analysis showed that Asp46 and Asp50, are highly conserved residues in the connexin family. They are positioned near the surface of the extracellular membrane facing the pore interior, and create a long, negatively-charged path. Along with the pore funnel, this region probably contributes to the charge selectivity and size restriction [44]. According to our model, when Asp50 is mutated several interactions are directly affected. This is so, becauseAsp50 forms the core of the inter-protomer interactions along with Gln48 and Asn62 in the extracellular domain from one protomer and Asp46 of the adjacent protomer. This might help to explain at the molecular level the pathogenicity of the mutation.

Collectively, this work presents the first report of a KID patient in Argentina. Moreover, it provides further insight into Cx26 structure-function and the underlying bases for the phenotypes associated with the p.Asp50Asn mutation. In addition, identification of the Asp50Asn mutation in *GJB2* allows the accurate classification of the skin disorder in the patient. Importantly, other syndromes that share common signs and symptoms can be ruled out and differential diagnosis from other childhood skin abnormalities can be effectively established. Early genetic diagnosis in patients with skin disorders and hearing impairment at birth allows early treatment and improvement of the quality of life.

### Consent

Written informed consent was obtained from the patient for publication of this Case report and any accompanying images. A copy of the written consent is available for review by the Editor of this journal.

## Abbreviations

1U: one unit
bp: base pair
CI: cochlear implant
Cx26: connexin26
dNTPs: deoxyribonucleotide triphosphate
Domains NT, TM1 to M4, E1- E2, CL, CT: amino-terminus, transmembrane one to four, extracellular domain one and two, cytoplasmic loop, carboxy terminus domain
*GJB2, GJB6*: gap junction beta 2 gene, gap junction beta 6 gene
kDa: kiloDaltons
KID: keratitis-ichthyosis-deafness
OMIM: online mendelian inheritance in man®
PCR: polymerase chain reaction
TEOAEs: transient evoked otoacoustic emissions
μM, mM, μl: microcolar, milimolar, microliter
